# Clinical evaluation of in-office bleaching with low, medium, and high concentrate hydrogen peroxide: a 6-month a double-blinded randomized controlled trial

**DOI:** 10.1007/s00784-025-06348-8

**Published:** 2025-04-21

**Authors:** Hanife Altınışık, Merve Nezir

**Affiliations:** Department of Restorative Dentistry, Faculty of Dentistry, Gazi University, Emek, 06510 Ankara, Turkey

**Keywords:** Bleaching efficacy, Hydrogen peroxide, In-office bleaching, Randomized clinical trial, Tooth sensitivity, Quality of life

## Abstract

**Objective:**

The study evaluated the longevity, efficacy, sensitivity, and impact on the oral health-related quality of life of in-office dental bleaching using low, medium, and high concentrate hydrogen peroxide.

**Material and Methods:**

Randomized, parallel, and double-blinded clinical trial was performed with 54 participants using18% hydrogen peroxide (HP), 25%HP, and 40%HP in-office bleaching agent. Tooth color was evaluated at baseline, after the 1st session bleaching, after the 2nd session bleaching and 6 months after finishing the bleaching using spectrophotometer. Tooth sensitivity (TS) was measured with the Visual Analog Scale at baseline, immediately after bleaching, after 1 day, and after 7 days. The impact on quality of life was evaluated using the 14-item Oral Health Impact Profile (OHIP-14) questionnaire at baseline, and 6 months after bleaching. The data were analyzed using the Kikare test, Kruskal Wallis test, one-way ANOVA, Wilcoxon, Friedman, Mann–Whitney (*p* < 0.05).

**Results:**

All groups achieved similar levels of bleaching during all evaluation times (*p* > 0.05), surpassing perceived value. There was no difference in TS levels among groups at all evaluation times (*p* > 0.05). TS significantly increased after bleaching (*p* < 0.05), decreased significantly after 24 h (*p* < 0.05), and there was no difference no difference between the initial sensitivity levels after 7 days (*p* > 0.05). All groups reported improved aesthetic self-perception following bleaching (*p* < 0.05) and there was no difference between the groups (*p* > 0.05).

**Conclusion:**

Low, medium, and high concentrations of HP did not affect both the final tooth color and the reported TS intensity, regardless of the evaluation time. In-office bleaching provides positive effects on aesthetic perception and different HP concentrations have not influenced this positive effect.

**Clinical significance:**

In this study, the efficacy, longevity, sensitivity, and impact on oral health-related quality of life of in-office bleaching using low, medium, and high concentration hydrogen peroxide agents from the same manufacturer were found to be similar. However, these results cannot be extrapolated to other in-office high-concentration hydrogen peroxide gels.

**Clinical trial registration number:**

NCT06700434.

## Introduction

In recent years, the significance of aesthetic appearance in dentistry has been increasing rapidly. Patients are seeking teeth that are not only straight but also white [1]. Discolored teeth may have a negative impact on an individual’s oral-health-related quality of life (OHRQoL) [2]. In-office bleaching is an effective and conservative clinical procedure that is commonly performed by dentists to solve aesthetic problems related to tooth discoloration [1, 3]. This aesthetic procedure is traditionally performed without the patient’s cooperation by using high concentrations of hydrogen peroxide (HP; 35% to 40%), a chemical with strong oxidative properties, producing results quickly [4, 5]. HP, which effectively whitens teeth by oxidizing organic components in the dental structure, can also easily penetrate the pulp chamber [6, 7]. This penetration is related to the pH of the bleaching gel, the concentration of HP, and the application time of this dental product on the tooth enamel [8]. HP can trigger tooth sensitivity (TS) [7] due to the activation of various inflammatory chemicals [9]. Post-bleaching TS may be experienced as a sudden, sharp pain or discomfort [5]. This sensitivity is temporary and typically disappears as the inflammatory response decreases [5, 10]. However, in some cases, it may cause patients to discontinue treatment [11].

The process of bleaching the teeth can impact individuals’ overall OHRQoL in various ways [12]. In many cases, bleaching is linked to positive outcomes, such as enhancing aesthetics and improving the appearance of the teeth, leading to a greater inclination to smile [13]. In contrast, the negative effects of bleaching may consist of pain, discomfort, and challenges in maintaining oral hygiene due to sensitivity or the irritation of the gums [14].

Several researchers have attempted to reduce post-bleaching TS by employing various treatment strategies. A few of these include administering pharmaceuticals (analgesics or anti-inflammatory drugs) [15], using desensitizing agents topically [16, 17] or within the bleaching agents [18], and selecting various HP concentrations [19, 20]. Therefore, in-office gels that are less concentrated than the widely used 35%-HP gel have been introduced to reduce the amount of HP reaching the pulp while maintaining the same level of bleaching efficacy. Certain clinical studies have investigated the effect of in-office gels with high versus medium concentrations of HP and high versus low concentrations of HP [21–23]. There is still some uncertainty regarding whether gels with high concentrations are more effective than others [6, 23]. This is because peroxide concentration as well as other characteristics of the bleaching gel, such as pH [8], viscosity [24], the type of thickener used [25], the humectant used, and the presence of pH adjusters [26] can affect the bleaching results. Therefore, this study tested the clinical effectiveness of various concentrations of hydrogen peroxide within the same formulation produced by the same manufacturer.

This randomized clinical study was intended to compare the bleaching efficacy, TS, and OHRQoL associated with in-office tooth bleaching procedures using varying concentrations of HP (low, medium, and high). The null hypotheses tested were as follows: (1) in-office tooth-bleaching procedures using varying concentrations of hydrogen peroxide (low, medium, and high) will have similar bleaching efficacy and longevity; (2) varying concentrations of hydrogen peroxide will lead to similar levels of TS after the bleaching procedures; and (3) at a six-month follow-up, there will be no difference in OHRQoL between the three bleaching systems tested.

## Materials and methods

### Ethical approval and protocol registration

**Fig. 1:**
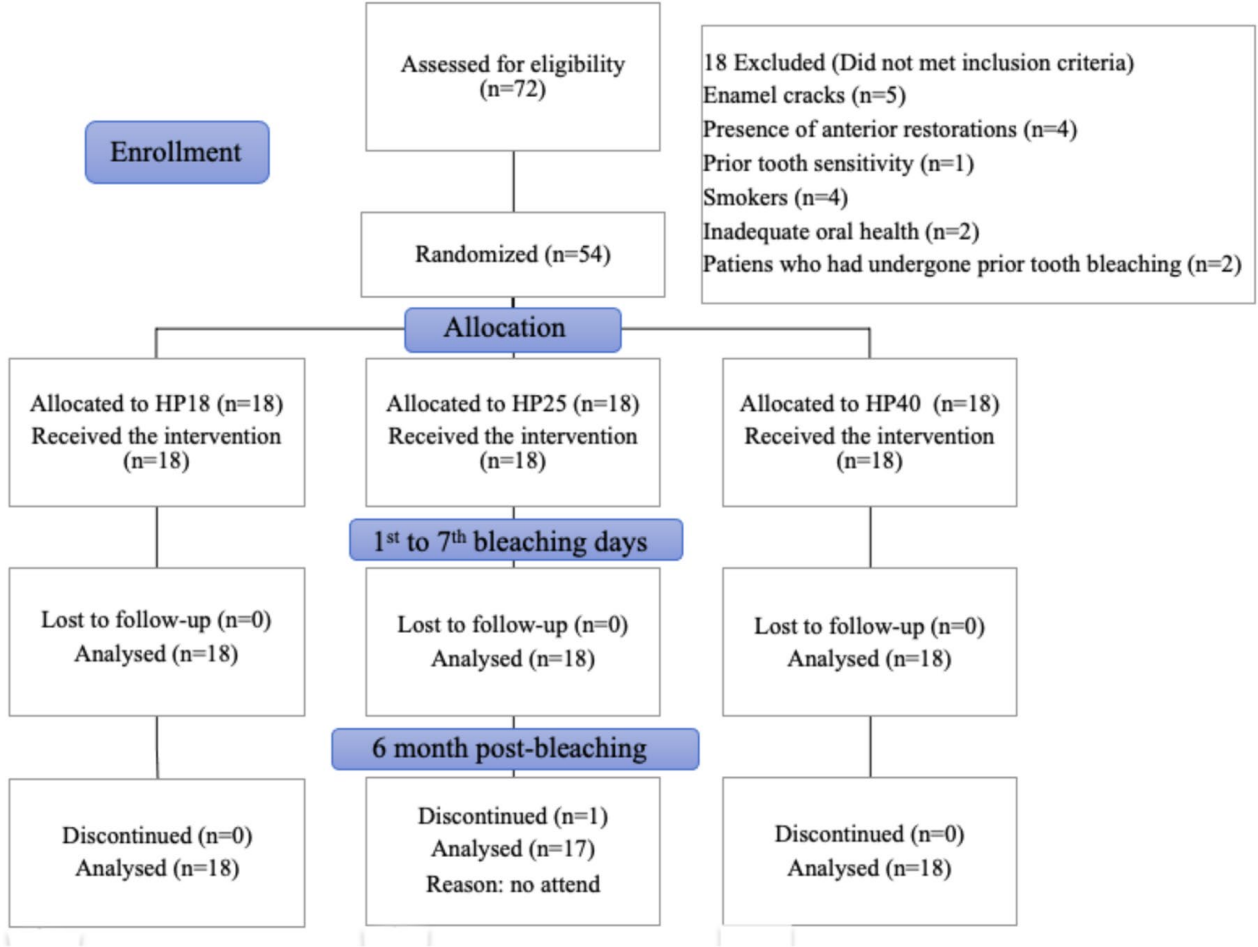
Flowchart detailing the protocol of this clinical trial

This clinical study adhered to the ethical guidelines outlined in the Helsinki Declaration and was approved by the Gazi University Faculty of Dentistry Clinical Research Ethics Committee (ethical protocol no. 2022.21/4). This study was registered at the US National Library of Medicine’s ClinicalTrials.gov website under reference number NCT06700434, and the findings were reported in compliance with the Consolidated Standards of Reporting Trials (CONSORT) Statement guidelines [27] (Fig. 1).

### Trial design, setting, and location of data collection

This study was a randomized, parallel, double-blind controlled clinical trial. It was performed from September 2023 to March 2024 in the Restorative Dentistry Clinic of the Gazi University Faculty of Dentistry.

### Recruitment

Participants were recruited on social media. The study was presented verbally to the participants, and they were informed about its aims. Participants who met the eligibility criteria and agreed to participate signed informed consent forms before being included in the study.

### Eligibility criteria

A total of 54 individuals were screened based on the specific requirements outlined in the inclusion and exclusion criteria. Males and females who were at least 18 years old and had good oral and general health, no periodontal disease, no caries, and teeth colored A3 or darker according to the VITA Easy Shade Guide (VITA Zanhnfabrik, BadSckingrn, Germany) were included. The exclusion criteria were as follows: having undergone a tooth-bleaching procedure in the previous two years, having tooth sensitivity, having used fixed orthodontic appliances or prosthetics on the maxillary and/or mandibular anterior teeth, having gingival recession, having parafunction, having discoloration due to tetracycline or fluorosis, smoking, and tooth fracture.

### Sample size calculation

The sample size for the study was determined using G*Power (Version 3.1.9.6) software based on a repeated measurement design. In this design, the treatment was considered the independent factor, and application time was the repeated measurement factor. With a significance level set at 5%, a statistical power of 80%, and an effect size of 24%, the required sample size was determined to be 15 participants per group. To account for an estimated 20% dropout rate, a total of 54 participants (18 participants per group) were recruited for the study.

### Random sequence generation and allocation concealment

The individuals who satisfied the eligibility requirements and agreed to participate were randomly selected and divided into three groups. Simple randomization was performed using a software program that is freely available online (https://www.graphpad.com/quickcalcs/randomize1.cfm) by someone external to the research protocol. During this randomization process, the details of the allocation into groups were recorded on cards contained in sequentially numbered, opaque, sealed envelopes. These envelopes were opened on the day of bleaching to prevent the disclosure of the randomization scheme.

### Blinding

The researcher responsible for data collection, the participants, and the statistician were blinded to the treatments assigned to the groups. Because the application procedures for the bleaching gels differed, the operator could not be blinded. The bleaching procedures were carried out by an operator, without revealing the concentration of the bleaching gels to the participants.

### Study intervention

The 54 volunteers selected were coded and then randomly divided into three groups (*n* = 18). One week before the bleaching treatment, the biofilm and external discoloration of all individuals were removed with prophylaxis, and they were provided with guidance regarding how to effectively maintain their oral hygiene. The bleaching procedures were performed by a single operator with more than four years of clinical experience, as described in CONSORT (Fig. 1). After the lip retractor (OptraGate, Ivoclar Vivadent, Schaan, Liechtenstein) was placed, the gingival barrier protection (Biowhiten, Biodent Ltd., İstanbul, Turkey) included with the products was applied to the upper and lower arches from premolar to premolar at the beginning of each session and removed after each treatment. The bleaching gels shown in Table 1 were applied according to the manufacturer’s instructions, removed after the recommended period with a disposable surgical saliva ejector, cleaned with gauze, and washed with air–water spray. Two bleaching sessions were performed, and there was a one-week interval between sessions. Participants were advised to maintain regular oral hygiene by brushing with toothpaste that did not contain desensitizing or whitening ingredients. The pH values of the bleaching gels used have been determined by the manufacturer and are shown in Table 1. To confirm the changes in the pH values of these gels during the process, they were placed in glass tubes with diameters of 15 mm, and their pH values were directly measured using a portable pH meter (HI 9321, Hanna Instruments, US). The device was calibrated with standard buffer solutions (Sigma, UK) at pH values of 4.0 and 7.0 prior to the analysis.

**Table 1:** Bleaching systems used in the study

Product, Manufacturer	Abbrevition	Application Protocol	Total Time	pH Indicated by the Manufacturer	Active Principle (Commercial Presentation)	Ingredients (Technical Profile)
Biowhiten, Biodent Ltd., İstanbul, Turkey	HP 40%	2 applications of 20 min per session	40 min	Alkaline (pH > 7)	Hydrogen Peroxide 40% and nano- hydroxyapatite	Water, Glycerin, Alcohol, Sodium bicarbonate, Sodium hydroxide, Hydrogen Peroxide 40% and nano- hydroxyapatite
Biowhiten, Biodent Ltd., İstanbul, Turkey	HP 25%	3 applications of 15 min per session	45 min	Alkaline (pH > 7)	Hydrogen Peroxide 25% and nano- hydroxyapatite	Water, Glycerin, Alcohol, Sodium bicarbonate, Sodium hydroxide, Hydrogen Peroxide 25% and nano- hydroxyapatite
Biowhiten, Biodent Ltd., İstanbul, Turkey	HP 18%	5 applications of 10 min per session	50 min	Alkaline (pH > 7)	Hydrogen Peroxide 18% and nano- hydroxyapatite	Water, Glycerin, Alcohol, Sodium bicarbonate, Sodium hydroxide, Hydrogen Peroxide 18% and nano- hydroxyapatite

### Color evaluation

Tooth color was evaluated objectively using a VITA Easy Shade V spectrophotometer (VITA Zanhnfabrik, BadSckingrn, Germany) initially, after the first bleaching, after the second bleaching, and 180 days after bleaching. The assesment was performed during daylight (10 a.m. to 2 p.m.) and by considering a 6-mm area situated in the middle section of the labial surface of the upper central incisors. To ensure consistency, alginate impressions were taken of the upper dental arches of each participant, and stone molds were created. Custom trays were fabricated using a 2-mm Essix plate (Essix C; Dentsply, FL, US) in a vacuum press machine (Ministar, Scheu, Iserlohn, Nordrhein-Westfalen, Germany). Six-mm-wide holes were drilled in the middle third of the buccal faces of the central incisors on these trays to serve as a guide when using Easyshade V.

After obtaining the L* (brightness), a* (green-to-red axis), and b* (blue-to-yellow axis) CIELab parameters from the spectrophotometer, color change was measured. The differences between baseline and post-bleaching-treatment Days 7 and 180 were calculated using the following CIELab formula [17]: ∆E_ab_ = [(∆L*)^2^ + (∆a*)^2^ + (∆b*)^2^]^1/2^. Additionally, color change was also calculated based on the CIEDE 2000 formula [17]: ∆E_00_ = [(ΔL/k_L_S_L_)^2^ + (ΔC/k_C_S_C_)^2^ + (ΔH/k_H_S_H_)^2^ + RT (ΔC × ΔH/S_C_ × S_H_)]^1/2^_._

### Tooth sensitivity evaluation

The participants were asked about the existence and level of TS or pain at baseline, immediately after the first bleaching session, 24 h later, and seven days later. To measure TS, the participants indicated their discomfort level using a visual analog scale (VAS) [28]. This scale is represented by a horizontal line measuring 10 cm in length. The endpoint of the line labeled “no pain” represents the absence of discomfort, while the opposite endpoint, labeled “worst pain,” represents the highest possible level of discomfort.

### Impact of oral condition on quality of life

The 14-item Oral Health Impact Profile (OHIP- 14) questionnaire (Table 2) was utilized to evaluate quality of life [2]. This questionnaire consists of seven domains and a total of 14 questions. Responses to these questions were recorded on a Likert scale based on participants’ answer choices. As options, the scale included never (0 points), rarely (1 point), sometimes (2 points), often (3 points), and always (4 points). A person’s OHRQoL is negatively affected when the average value of the seven domains is higher.

**Table 2:** Domains and questions in the OHIP- 14

OHIP- 14 questionnaire	
Domain	Questions
Functional limitation	Have you noticed a tooth that does not look right? Have you felt that your appearance has been affected by problems with your teeth?
Physical pain	Have you had sensitive teeth for example to heat or cold food or drinks? Have you had painful areas in your mouth?
Psychological discomfort	Have you been self-conscious because of your teeth? Have you felt uncomfortable about the appearance of your teeth?
Physical disability	Have you felt that your food is less tasty because of problems with your teeth? Have you avoided smiling because of problems with your teeth?
Psychological disability	Have you found it difficult to relax because of problems with your teeth? Have you been a bit embarrassed because of problems with your teeth?
Social disability	Have you been less tolerant of your spouse or family because of problems with your teeth? Have you had difficulties doing your usual job because of problems with your teeth?
Handicap	Have you been unable to enjoy the company of other people very much because of problems with your teeth? Have you felt that life in general was less satisfying because of problems with your teeth?

### Statistical analysis

The statistician was blinded to the groups. The data were analyzed with a statistical analysis program (IBM SPSS 25, V. 27.0, IBM, Chicago, IL). According to the results of a Shapiro–Wilk test, appropriate methods were used in this study. Parametric tests were used when a normal distribution was found for the groups, while non-parametric tests were used when a normal distribution was not found. The groups were compared in terms of gender using the chi-squared test. Kruskal–Wallis and one-way ANOVA tests were used to compare the groups in terms of age and measurements. Mann–Whitney, Tukey, Friedman, and Wilcoxon tests were used to compare individuals within groups in terms of measurements.

## Results

### Characteristics of the included participants

Seventy-two individuals were assessed, and 54 were included in the clinical trial. After six months, only one individual in the HP25 group failed to attend their follow-up appointment, and 53 patients completed the treatment (Fig. 1). The baseline features of the groups are shown in Table 3. The mean age values of the participants in the groups were similar (HP18: 22.33 ± 1.03 years; HP25: 22.28 ± 1.07 years; HP40: 22.22 ± 1.73 years). Among the participants, 61.1%, 50%, and 55.6% of those from the HP18, HP25, and HP40 groups, respectively, were male. No variables were found to significantly differ between the groups (*p* > 0.05).

**Table 3:** Participant baseline characteristics

Baseline features	Groups			*p*-value
	HP18 (*n* = 18)	HP25 (*n* = 18)	HP40 (*n* = 18)	
Age (years; mean ± SD)	22,33 ± 1,03	22,28 ± 1,07	22,22 ± 1,73	0,589^1^
Minumum age (years)	21	21	21	
Maximum age (years)	24	24	28	
Male (%)	61,1	50	55,6	0,799^2^
L* (mean ± SD)	80,85 ± 4,8	81,81 ± 3,98	80,34 ± 4,6	0,609^3^
a* (mean ± SD)	0,4 ± 0,5	0,26 ± 0,95	0,21 ± 0,99	0,401^3^
b* (mean ± SD)	22,28 ± 0,17	22,81 ± 4,24	21,78 ± 2,79	0,429^1^

SD standard deviation, *p < 0,05, ^1^Kruskal Wallis test, ^2^Kikare test, ^3^One-way ANOVA L*, a* and b*. L*stands for the lightness, and a* and b* for the green–red and blue–yellow axis of the CIElab color space, respectively

### Color change

Table 4 illustrates the post-bleaching color evaluation results for the three groups, which had similar mean L, a, and b values initially, as shown in Table 3. No differences were observed between the groups at any point in the assessment of both ∆E_ab_ and ∆E_00_ (*p* > 0.05), and after the first in-office session, significant whitening was observed in all three groups (*p* > 0.05). According to ∆E_ab_, the second in-office session provided a significant additional bleaching effect in groups HP18 and HP25 (*p* < 0.05), while the bleaching effect in group HP40 was not statistically significant (*p* > 0.05). According to ∆E_00_, the bleaching effect of the second in-office session was not significant in any of the three groups (*p* > 0.05). A significant color rebound effect was observed in all three groups after 6 months according to both ∆E_ab_ and ∆E_00_ (*p* < 0.05).

**Table 4:** Statistical analysis of the color change expressed in ΔE units (mean and standard deviation) at all time points

Color change instrument	Time assesments	Groups			^1,2^*p*-value
		HP18	HP25	HP40	
	Baseline vs. post- bleaching	4,96 ± 3,70^a^	5,42 ± 3,45^a^	6,05 ± 3,10^a^	0,371^1^
∆E_ab_	Baseline vs. 1 week after bleaching	6,50 ± 2,64^b^	6,92 ± 2,86^b^	7,09 ± 3,92^a^	0,845^2^
	Baseline vs. 6 months after bleaching	3,37 ± 2,53^c^	3,44 ± 3,15^c^	3,49 ± 2,29^c^	0,786^1^
	^**3**^**p**	0,000*	0,000*	0,002*	
	Baseline vs. post- bleaching	3,20 ± 2,66^a^	3,90 ± 2,01^a^	4,09 ± 3,54^a^	0,327^1^
∆E_00_	Baseline vs. 1 week after bleaching	3,81 ± 1,73^a^	4,35 ± 2,56^a^	4,78 ± 2,37^a^	0,459^1^
	Baseline vs. 6 months after bleaching	2,29 ± 1,99^b^	2,31 ± 1,66^b^	2,54 ± 2,87^b^	0,801^1^
	^**3**^**p**	0,012*	0,006*	0,001*	

^1^Kruskal Wallis, ^2^One-way ANOVA, ^3^Friedman test *p < 0,05

Different letters in the columns indicate the difference between groups

### Tooth sensitivity evaluation

A statistical comparison of TS intensity at various assessment points for all three groups is presented in Table 5. No differences were observed among the groups at any point during the assessment (*p* > 0.05). TS was transient up to 24 h and was not recorded at seven days post-procedure.

**Table 5:** Statistical comparison of tooth sensitivity intensity at different assessment points for all three groups

Assessment times	Groups			^1,2^*p*-value
	HP18	HP25	HP40	
T_0_ (Before treatment)	0,2 ± 0,19^a^	0,21 ± 0,25^a^	0,2 ± 0,23^a^	0,977^1^
T_1_ (After treatment)	0,86 ± 0,61^b^	1,06 ± 0,62^b^	1,32 ± 0,63^b^	0,072^1^
T_2_ (24 h)	0,59 ± 0,34^c^	0,65 ± 0,28^c^	0,79 ± 0,44^c^	0,257^2^
T_3_ (7 day)	0,25 ± 0,19^a^	0,29 ± 0,16^a^	0,34 ± 0,24^a^	0,494^1^
^**3**^***p*-value**	0,000*	0,000*	0,000*	

^1^Kruskal Wallis, ^2^One-way ANOVA, ^3^Friedman test *p < 0,05

Different letters in the columns indicate the difference between groups

### Impact of oral condition on quality of life

The statistical analysis of the mean OHIP scores before and 6 months after the bleaching treatment is shown in Table 6. The mean ± standard deviation for baseline overall OHIP- 14 scores for participants in the HP18, HP25, and HP40 groups were 15.67 ± 9.72, 15.33 ± 5.58, and 14.17 ± 7.25, respectively. At the 6-month follow-up, significant differences in overall OHIP- 14 scores were observed in all groups as compared to baseline levels (*p* < 0.05). When evaluating improvements in individual domains within groups, all groups showed significant improvements regarding functional limitations, psychological discomfort, physical disability, psychological disability, and social disability (*p* < 0.05). Furthermore, there were no significant differences between groups in terms of overall OHIP- 14 scores or the scores for any domain at baseline or after treatment (*p* > 0.05).

**Table 6:** Mean OHIP scores and standard deviation (SD) before and 6 months after bleaching treatment

Domains	Assessment times	Groups			*p*-value
		HP18 Mean ± SD	HP25 Mean ± SD	HP40 Mean ± SD	
Functional limitation	Baseline	1,19 ± 0,64	1,17 ± 0,45	1,14 ± 0,87	0,958^1^
	After Bleaching	0,92 ± 0,65	0,83 ± 0,24	0,89 ± 0,68	0,871^1^
	^3^*p*-value	0,026*	0,010*	0,024*	
Physical pain	Baseline	1,00 ± 0,45	0,92 ± 0,67	0,94 ± 1,16	0,524^1^
	After Bleaching	0,78 ± 0,31	0,67 ± 0,49	0,69 ± 0,52	0,738^1^
	^3^*p*-value	0,159	0,086	0,473	
Psychological discomfort	Baseline	1,69 ± 0,79	1,67 ± 0,45	1,61 ± 0,70	0,880^1^
	After Bleaching	0,81 ± 0,71	0,75 ± 0,49	0,75 ± 0,77	0,919^1^
	^3^*p*-value	0,003*	0,001*	0,005*	
Physical disability	Baseline	1,31 ± 0,62	1,33 ± 0,51	1,22 ± 0,69	0,906^1^
	After Bleaching	0,53 ± 0,58	0,53 ± 0,44	0,50 ± 0,30	0,902^1^
	^3^*p*-value	0,001*	0,002*	0,007*	
Psychological disability	Baseline	0,72 ± 0,91	0,75 ± 0,67	0,78 ± 0,49	0,557^1^
	After Bleaching	0,28 ± 0,26	0,33 ± 0,34	0,31 ± 0,52	0,680^1^
	^3^*p*-value	0,014*	0,004*	0,004*	
Social disability	Baseline	0,42 ± 0,52	0,47 ± 0,56	0,44 ± 0,45	0,928^1^
	After Bleaching	0,17 ± 0,38	0,19 ± 0,35	0,11 ± 0,27	0,695^1^
	^3^*p*-value	0,007*	0,026*	0,010*	
Handicap	Baseline	0,39 ± 0,40	0,42 ± 0,39	0,36 ± 0,48	0,805^1^
	After Bleaching	0,31 ± 0,39	0,31 ± 0,35	0,28 ± 0,46	0,795^1^
	^3^*p*-value	0,257	0,102	0,083	
Total score	Baseline	15,67 ± 9,72	15,33 ± 5,58	14,17 ± 7,25	0,829^2^
	After Bleaching	10,58 ± 4,03	12,44 ± 4,84	10,25 ± 2,90	0,274^1^
	^3^*p*-value	0,019*	0,035*	0,026*	

^1^Kruskal Wallis, ^2^One-way ANOVA, ^3^Wilcoxon *p < 0,05

## Discussion

HP is the key ingredient in bleaching gels that causes bleaching by reacting with organic structures via reactive oxygen species. These species target large, dark-colored chromophore molecules, breaking them down into smaller, less pigmented molecules [29]. Kose et al. [30] report that the degree of color change is influenced by gel concentration, application time, and the number of times the gel is renewed on the tooth surface. One study reported that bleaching efficiency increases as the HP concentration in the bleaching gel used increases because the number of free radicals in peroxide solutions is related to HP concentration [31]. However, some clinical studies investigating the efficacy of office gels with high- (> 30%) versus medium-concentration HP (20 ≥ HP ≤ 30) and high- versus low-concentration (< 20%) HP have reported that the bleaching effect is not purely concentration dependent [22, 32, 33].

Paravina et al. [34] report that the 50:50% perceptibility threshold and 50:50% acceptability threshold values were 1.2 and 2.7, respectively, in the CIELab system, and 0.8 and 1.8, respectively, in the CIEDE2000 system. The objective color change assessment demonstrated that all three groups experienced effective and clinically perceptible tooth whitening. The first null hypothesis investigated in this study was accepted, as the low- and medium-concentration HP used for in-office bleaching showed efficacy levels similar to that of high-concentration HP during the evaluated periods (Table 4). The results indicate that there were no significant differences between the three groups. In this clinical study, the application times of the 40%-HP, 25%-HP, and 18%-HP bleaching agents were 40, 45, and 50 min, respectively, with the number of renewals on the tooth surface being two, three, and five, respectively. This finding is aligned with Sulieman et al. [35] and Monteiro et al. [36], who state that all bleaching agents were effective, regardless of HP concentration, but that the number of applications required did depend on HP concentration.

One of the most common side effects observed during and after bleaching is TS, which occurs due to the ability of HP to penetrate the tooth structure and cause an inflammatory response in the pulp [37, 38]. It has been reported that the higher the HP concentration, the greater the risk and intensity of TS for patients [28, 39]. In addition, longer application times and an increased number gel renewals on the tooth surface increase HP penetration [40]. Due to this common side effect, bleaching gels containing various concentrations of HP and remineralizing agents, such as nano-hydroxyapatite (n-HAP), have been introduced to eliminate or, at least, reduce the risk of bleaching-induced TS. n-HAP is widely used in various therapeutic applications, including remineralization and reducing sensitivity. It has been reported that the microscopic surface enamel defects caused by the bleaching process can be repaired using a special paste containing n-HAP crystals [41]. Due to its nano-scale dimensions, n-HAP has the ability to effectively infiltrate dentinal tubules and enamel microcracks, leading to the secure sealing of these structures. Furthermore, n-HAP aids in the restoration of both the microstructure and chemical composition of tooth tissues [42, 43]. Browning et al. [44] report that the application of a paste containing n-HAP successfully decreased hypersensitivity following bleaching procedures. Ferraz et al. [42] report that various concentrations of nHA in 35%-HP bleaching gel did not interfere with bleaching efficacy for the enamel and deep dentin and did not affect enamel bond strength after bleaching. A systematic review and meta-analysis demonstrate that desensitizing agents containing n-HAP are effective in reducing dentin hypersensitivity in both at-home and in-office treatments as compared to other desensitizing agents or placebo/negative control [45]. Vano et al. [43] report that using a 6%HP bleaching gel containing 2% n-HAP reduced sensitivity during bleaching treatments as compared to a 6%HP bleaching gel without n-HAP. The present study investigated bleaching gels with low, medium, and high HP concentrations that also contained n-HAP.

The manufacturer claims that the bleaching gels used in this study maintain high, stable pH values during the bleaching process. This claim has been verified by the researchers via pH measurements. The rate at which hydrogen peroxide decomposes and the types of by-products it generates are influenced by the pH level of the solution. It has been reported that HP is more efficient in terms of bleaching when applied in an alkaline environment [46]. Clinical trials have indicated that when gels with a neutral or alkaline pH are used, the intensity and severity of sensitivity are reduced as compared to when gels with an acidic pH are used [47, 48]. Kwon et al. [24] found that the viscosity of bleaching gel had no impact on effectiveness but that gels with low viscosity enhanced the the penetration of HP. Among the in-office bleaching gels used in this study, the 25%-HP and 18%-HP gels had lower viscosity than the 40%-HP gel. The 25%-HP and 18%-HP gels may have penetrated more deeply into the tooth due to their lower viscosity, application time, and number of changes as compared to the 40%-HP gel. For these reasons, the second null hypothesis investigated in this study was also accepted, as the high-concentration HP used for in-office bleaching led to TS levels similar to those of the low- and medium-concentration HP during the evaluated periods.

The OHIP- 14 is a widely used and trustworthy tool for evaluating OHRQoL results, and its validity has been confirmed across various age ranges and in several countries [49]. The findings of this study show that all the tested bleaching protocols, regardless of HP concentration (low, medium, or high), were associated with a significant improvement in OHRQoL based on the OHIP- 14. However, because there were no differences in OHRQoL between the three bleaching systems tested at a six-month follow-up, the third null hypothesis was accepted. This finding is consistent with those of other studies, despite the use of various bleaching protocols across them [1, 2, 13, 14, 50]. Bersezio et al. [13] suggest that when tooth whitening is maintained, patients tend to experience positive changes in their psychological well-being, social interactions, and overall functionality. In the present study, all groups also experienced a significant improvement in the functional limitations, psychological discomfort, physical disability, psychological disability, and social disability domains. The findings show that all the examined vital teeth bleaching procedures significantly enhanced an individual’s self-assessment of their dentofacial appearance.

This study has certain limitations that must be considered. One is the fact that the majority of participants were young adults, which may limit the generalizability of the findings to a wider population. Another is the fact that the subjective nature of sensitivity and quality-of-life reporting could have influenced the observed results.

## Conclusion

In-office bleaching treatments with low-, medium-, and high-concentration HP agents obtained from the same manufacturer were found to be similar in terms of their effectiveness, longevity, TS intensity, and effect on OHRQoL. In addition, a positive psychosocial effect was measured in patients six months after the bleaching treatment as compared to the beginning of the treatment. Thus, the treatment had a positive effect on the patients’ self-esteem and, ultimately, their OHRQoL.

## Data Availability

The datasets generated and analyzed in the current study are available from the corresponding author upon reasonable request.
